# Cardiovascular disease guideline adherence and self-reported statin use in longstanding type 1 diabetes: results from the Canadian study of longevity in diabetes cohort

**DOI:** 10.1186/s12933-015-0318-9

**Published:** 2016-01-25

**Authors:** Johnny W. Bai, Geneviève Boulet, Elise M. Halpern, Leif E. Lovblom, Devrim Eldelekli, Hillary A. Keenan, Michael Brent, Narinder Paul, Vera Bril, David Z. I. Cherney, Alanna Weisman, Bruce A. Perkins

**Affiliations:** Division of Endocrinology and Metabolism, Department of Medicine, University of Toronto, Leadership Sinai Centre for Diabetes, Mount Sinai Hospital, L5-210, 60 Murray Street, Mail Box 16, Toronto, ON M5T 3L9 Canada; Research Division, Joslin Diabetes Center, Boston, MA USA; Department of Ophthalmology and Vision Sciences, Department of Medicine, University of Toronto, Toronto, ON Canada; Joint Department of Medical Imaging, Division of Cardiothoracic Radiology, University Health Network, Toronto, ON Canada; The Ellen and Martin Prosserman Centre for Neuromuscular Diseases, Krembil Neuroscience Centre, Division of Neurology, Department of Medicine, University Health Network, University of Toronto, Toronto, ON Canada; Division of Nephrology, Department of Medicine, University of Toronto, Toronto, ON Canada

**Keywords:** Type 1 diabetes, Cardiovascular disease, HMG-CoA reductase inhibitor (3-hydroxy-3-methyl-glutaryl-CoA reductase), Statin, Adherence

## Abstract

**Background:**

Older patients with longstanding type 1 diabetes have high cardiovascular disease (CVD) risk such that statin therapy is recommended independent of prior CVD events. We aimed to determine self-reported CVD prevention guideline adherence in patients with longstanding diabetes.

**Research design and methods:**

309 Canadians with over 50 years of type 1 diabetes completed a medical questionnaire for presence of lifestyle and pharmacological interventions, stratified into primary or secondary CVD prevention subgroups based on absence or presence of self-reported CVD events, respectively. Associations with statin use were analyzed using multivariable logistic regression.

**Results:**

The 309 participants had mean ± SD age 65.7 ± 8.5 years, median diabetes duration 54.0 [IQR 51.0, 59.0] years, and HbA1c of 7.5 ± 1.1 % (58 mmol/mol). 159 (52.7 %) participants reported diet adherence, 296 (95.8 %) smoking avoidance, 217 (70.5 %) physical activity, 218 (71.5 %) renin-angiotensin-system inhibitor use, and 220 (72.1 %) statin use. Physical activity was reported as less common in the secondary prevention subgroup, and current statin use was significantly lower in the primary prevention subgroup (65.5 % vs. 84.8 %, p = 0.0004). In multivariable logistic regression, the odds of statin use was 0.38 [95 % CI 0.15–0.95] in members of the primary compared to the secondary prevention subgroup, adjusting for age, sex, hypertension history, body mass, HbA1c, cholesterol, microvascular complications, acetylsalicylic acid use, and renin-angiotensin system inhibitor use.

**Conclusion:**

Despite good self-reported adherence to general CVD prevention guidelines, against the principles of these guidelines we found that statin use was substantially lower in those without CVD history. Interventions are needed to improve statin use in older type 1 diabetes patients without a history of CVD.

## Background

Cardiovascular disease (CVD), which includes myocardial infarction, coronary artery disease (CAD), stroke, and peripheral vascular disease, is often cited as the primary cause of mortality in type 1 diabetes mellitus [1–4]. Though it has been suggested that people with diabetes have a two to fourfold excess risk of developing CVD, in the context of type 1 diabetes the magnitude of this risk approaches tenfold [5–7]. The etiology for amplification of lifetime CVD risk in type 1 diabetes may relate to longer duration of exposure to hyperglycemia [7, 8] in part owing to relatively younger age at diagnosis. Thus, older patients with long duration of type 1 diabetes are a unique group with extremely high lifetime risk of CVD.

More intensive cardiovascular protection measures are recommended for older patients with longstanding diabetes regardless of their CVD history, particularly with regard to pharmacotherapy for lipid control [9, 10]. Clinical guidelines on vascular protection in diabetes recommend the use of 3-hydroxy-3-methylglutaryl-coenzyme A reductase inhibitors (commonly referred to as “statins”) in patients who are over age 40, have long duration of diabetes, or are younger but have existing microvascular complications or additional risk factors [10–12]. The benefits of statin use and lowering LDL cholesterol (LDL-C) in diabetes are strongly supported by studies which demonstrate the effectiveness of statins in reducing risk of vascular events and mortality regardless of prior CVD history [13, 14]. Other general strategies for vascular protection include smoking cessation, dietary modification, regular physical exercise, maintenance of optimal glycemic control, blood pressure, and weight, and the use of renin-angiotensin system (RAS) inhibitors such as angiotensin-converting enzyme inhibitors (ACEi) or angiotensin receptor blockers (ARB). The evidence that justifies acetylsalicylic acid use is focused mainly on secondary rather than primary CVD prevention [11, 12]. Ultimately, in the context of long diabetes duration and older age, a key emphasis of guidelines is the use of pharmacotherapy—in particular statin therapy—independent of CVD history.

Despite strong evidence for CVD primary prevention strategies, studies in general practice settings have noted low adherence to these guidelines, possibly due to the nature of preventative rather than therapeutic interventions, expense, concerns about efficacy and side-effects, and limitations in physician-patient relationships [15–18]. Suboptimal statin adherence has been shown in CVD primary prevention (those without history of CVD), with long duration of therapy, and in elderly patients. Under-treatment substantially increases the risk of adverse cardiovascular outcomes and mortality [18–23]. To our knowledge, there are no studies which examine attention to CVD prevention guidelines, and specifically statin use, in patients with longstanding type 1 diabetes.

We aimed to determine whether guideline adherence and self-reported statin use differed between those with and without history of CVD in the baseline phase of the Canadian Study of Longevity in Type 1 Diabetes cohort consisting of patients with 50 years or more of type 1 diabetes at uniformly high risk of CVD. Disparity in the comparison between those with and without CVD history may indicate suboptimal implementation of current clinical practice guidelines and a disregard for longstanding diabetes as a fundamental CVD risk factor.

## Research design and methods

### Study overview

This study was conducted as a secondary analysis of the baseline data from the Canadian Study of Longevity in Type 1 Diabetes cohort (JDRF operating grant 17-2013-312). The goal of this analysis was to describe adherence to CVD prevention guidelines—with an emphasis on self-reported statin use—in Canadians living with type 1 diabetes for 50 years or more.

### Participant recruitment

**Fig. 1:**
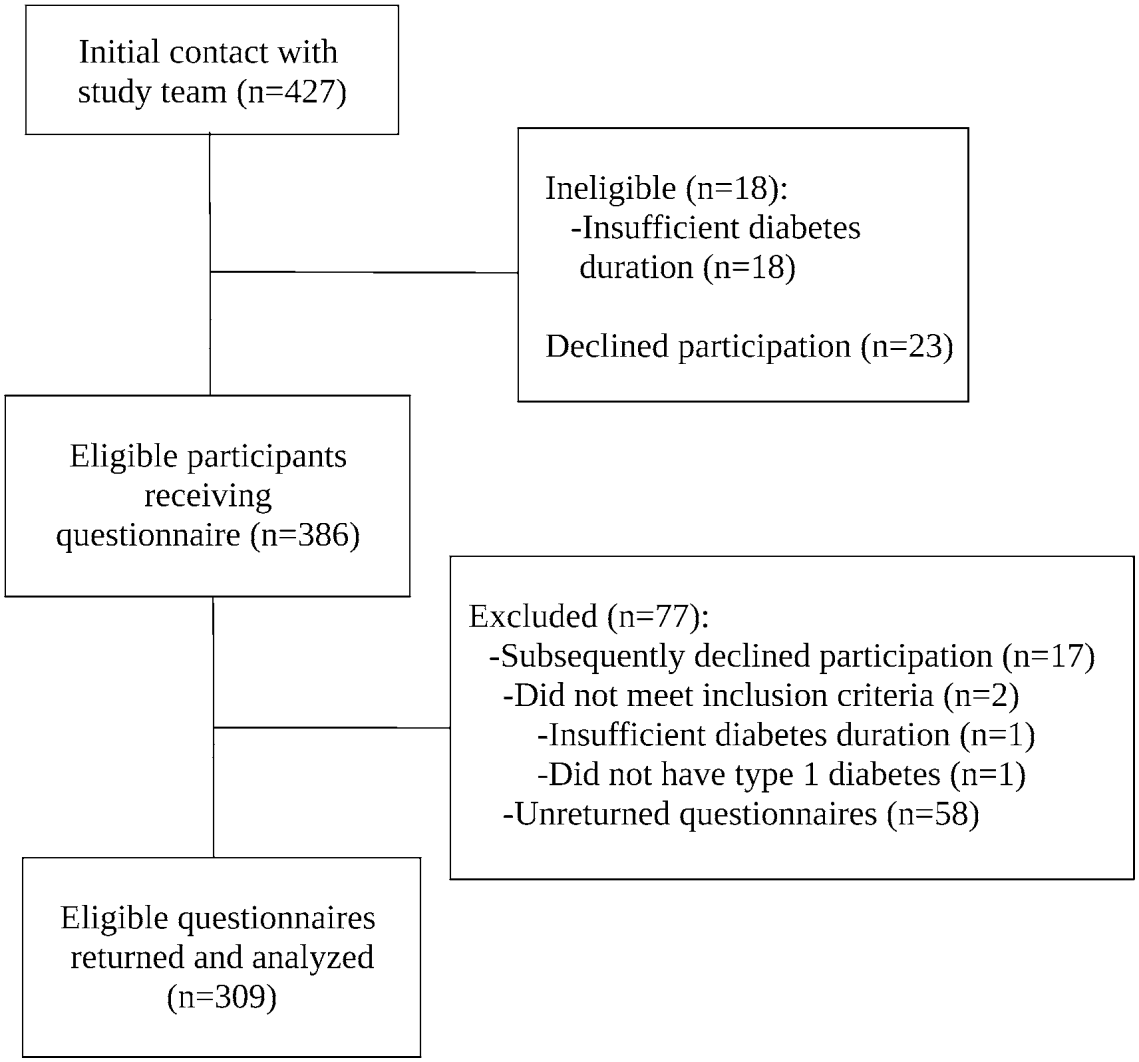
Participant flow diagram

Between April 2013 and December 2014, patients were contacted across Canada through public advertisements, social media, and mailings to health care professionals including primary care physicians, endocrinologists, and pharmacists. Akin to other cohorts, our study included patients with a history of at least 50 years of insulin dependence, as acknowledged through medical documentation or corroboration by a family member [24, 25]. For the Canadian Study of Longevity in Type 1 Diabetes, we anticipated a total cohort sample size of approximately 300 participants based on Canadian 1962 census data and contemporaneous age-specific incidence rates of type 1 diabetes and survival curves [26, 27]. A total of 427 people initially contacted us by toll free number, mail, or e-mail, and 386 eligible participants agreed to participate. By the time of analysis, 309 questionnaires were returned and these participants were included in analysis. Participant flow is summarized in Fig. 1. Participant recruitment and data entry remains ongoing. Participants provided written informed consent, and the study protocol was approved by the ethics committee of the Mount Sinai Hospital (Toronto, ON, Canada).

### Data collection

Data were collected through a 35-page questionnaire in which participants were asked about their diabetes management, family history of CVD, lifestyle and smoking habits, medication use, and history of cardiovascular disease (angina and heart attack), related surgeries (cardiac/leg bypass and angioplasty), hypertension and other medical history including cerebrovascular disease. Furthermore, we obtained from participants’ healthcare providers recent clinical, physical, and laboratory measurements including blood pressure, lipid profile, HbA1c, estimated glomerular filtration rate (eGFR), and fundoscopy examination results.

Lifestyle variables included questions related to diet, smoking, and physical activity: Diet was assessed using seven questions surrounding nutrient and caloric intake, meal patterns, and meal content. Dietary adherence was defined by presence of (1) self-reported consumption of fruits and vegetables and (2) self-reported effort to moderate consumption of dietary carbohydrates and fats. Participants reported presence or absence of current smoking and physical activity, and provided body weight and height from which BMI was calculated. To define pharmacotherapy use, participants were asked to list all current medications, allowing determination of acetylsalicylic acid, statin, ezetimibe, fibrate, and RAS inhibitor use; history of side-effects, drug intolerance, and duration of therapy were not ascertained. As participants were over the age of 40, according to guidelines all participants were eligible for statin use [10–12]. Also according to guidelines, RAS inhibitor use is indicated for participants older than 55 years, with microvascular complications, or with prior CVD history. Subjects meeting these criteria and reporting use of RASi were considered to be adherent [11].

For descriptive purposes, an “adherence index” was created based on self-reported clinical variables and laboratory results, compared to clinical practice guideline recommendations [10–12]. Participants were assessed for attainment of (1) recommended diet; (2) lack of smoking; (3) reported physical activity; (4) glycemic control [HbA1c ≤7 % (53 mmol/mol)]; (5) blood pressure control (≤130/80 mmHg); (6) LDL-C ≤2.0 mmol/L; (7) maintenance of optimal body weight (BMI <25.0 kg/m^2^); (8) self-reported use of statin therapy for participants ≥40 years of age (i.e. all study participants); (9) self-reported RAS inhibitor therapy for secondary prevention, age ≥55 years, or age <55 years but with microvascular complications. Adherence was reported as frequency and proportion of participants who attained each recommendation. These recommendations were stratified into domains of lifestyle adherence (recommendations 1–3), clinical target adherence (recommendations 4–7), and pharmacotherapy use (recommendations 8–9). As acetylsalicylic acid use for primary prevention is not unanimously supported by evidence, it was not used in the adherence index.

Nephropathy was defined by the presence on laboratory tests of albumin to creatinine ratio (ACR) ≥2 mg/mmol or an age-adjusted glomerular filtration rate (GFR) <60 ml/min [28]. Presence of retinopathy, and its classification as proliferative or non-proliferative, was obtained by the recent eye specialist examination. Presence of symptomatic diabetic neuropathy was determined through the use of the 15-item, self-administered Michigan Neuropathy Screening Instrument (MNSI) questionnaire. Neuropathy was defined by a score ≥3 [29].

### Primary and secondary prevention subgroups

Participants were stratified into primary and secondary prevention subgroups for comparison. The secondary prevention subgroup consisted of participants who reported any previous diagnosis of coronary artery disease, heart attack or angina, history of cardiac or leg angioplasty, bypass graft surgery, or cerebrovascular disease including stroke. Participants without any of these factors were considered to be in the primary prevention subgroup. As this study was a secondary analysis of cohort data, there were no specific questions about cerebrovascular incidents; history of such events was determined from an open-ended question for participants to report all known medical conditions and history.

### Statistical analysis

SAS version 9.2 (SAS Institute, Cary, NC, USA) was used to perform statistical analysis. Descriptive characteristics were reported as mean ± standard deviation (SD), median and interquartile range (IQR), or as frequency and percent. Statistical comparisons between primary and secondary prevention subgroups were made using the Student’s *t* test, the Mann–Whitney U test, or the χ^2^-test, depending on the distribution of the variable. For the adherence index, the χ^2^-test was used to compare adherence rates between primary and secondary prevention subgroups; Cohen’s kappa coefficient was used to assess agreement among each index. Logistic regression was performed to assess the association of CVD history with self-reported statin use: first univariable models were used to identify other participant characteristics that were significantly associated with statin use. In order to adjust for these potential confounders, these characteristics were then included as independent variables along with CVD history in a final multivariable model, with statin use as the dependent variable. Age, sex, and HbA1c were included a priori, as well as all significant predictors (p < 0.05) in univariable analyses. Multicollinearity among the independent predictor variables was assessed. Odds ratios (OR) are reported along with their 95 % confidence intervals. As a sensitivity analysis, the multivariable model was also run using a stepwise variable selection model. As a second sensitivity analysis, the logistic regression was restricted to the primary prevention subgroup. P-values < 0.05 were considered statistically significant. The sample size was estimated to have a power of 0.81 to detect at least a 15 % difference in proportion of statin use between primary and secondary prevention subgroups, based on the assumptions of approximately 50 % statin use [22] and 50 % prevalence of CVD in longstanding type 1 diabetes [24].

Missing data was assumed to be missing at random. Systolic blood pressure (SBP) and diastolic blood pressure (DBP) data was incomplete in that 150(49 %) of values were unreported in the physical exam reports from health care providers. For this reason, self-reported history of hypertension was instead used in the multivariable model, but we performed a sensitivity analysis using SBP. Available-case analysis was used to report patient characteristics, guideline adherence values, and univariable screening, whereas complete-case analysis was used for multivariable regression. To honour variations in threshold values reported by different international organizations that provide clinical practice guidelines, a sensitivity analysis was performed using higher HbA1c of 7.5 % (58 mmol/mol), 8.0 % (64 mmol/mol), and 8.5 % (69 mmol/mol), blood pressure <140/90 mmHg, and BMI <30 kg/m^2^ targets as cut-offs [12, 30, 31]. Furthermore, as some guidelines recommend special considerations for statin use for patients of extreme age, we performed sensitivity analysis by comparing statin use only among participants who were 75 years or younger [12].

## Results

Patient characteristics are summarized in Table 1 according to the total cohort, primary prevention, and secondary prevention subgroups. The 309 participants had a mean ± SD age of 65.7 ± 8.5 years and median diabetes duration of 54.0 [IQR 51.0, 59.0] years, with 137 (44.5 %) participants being male and 13 (4.2 %) non-Caucasian. Notably, the primary prevention subgroup was significantly younger (64.3 ± 8.5 vs. 68.5 ± 8.3 years, p < 0.001), had shorter diabetes duration (53.0 [50.0, 57.0] vs 57.0 [52.5, 60.5] years, p < 0.001), and contained a smaller proportion of males (82.0 (40.4 %) vs. 55 (52.4 %), p = 0.045). There were no significant differences between primary and secondary prevention subgroups in elements of the family history. Clinical features are shown in the second section of the table. The 309 participants had a BMI of 25.0 [23.0, 28.2], SBP of 128.8 ± 14.9 mmHg, and DBP of 67.4 ± 8.9 mmHg, and 182 (60.3 %) reported history of hypertension. Of these clinical variables, relative to the secondary prevention subgroup, the primary prevention subgroup had a lower proportion of hypertension history (111 (56.1 %) vs. 71 (68.3 %), p = 0.04). For laboratory report values, the whole cohort had HbA1c of 7.5 ± 1.1 % (58 mmol/mol), total cholesterol of 4.1 ± 0.9 mmol/L, and LDL-C of 2.0 ± 0.7 mmol/L. The primary prevention subgroup had lower HbA1c (7.4 ± 1.1 (57 mmol/mol) vs. 7.7 ± 1.1 % (61 mmol/mol), p = 0.04), higher total cholesterol (4.24 ± 0.92 vs. 3.95 ± 0.86, p = 0.02), lower triglycerides (0.77 [0.60, 1.00] vs. 0.83 [0.66, 1.23] mmol/L, p = 0.04), and higher HDL-C (1.79 ± 0.52 vs. 1.58 ± 0.49 mmol/L, p = 0.002). The two subgroups did not differ significantly in LDL-C. Regarding diabetes complications, 193 (71.5 %) had retinopathy, 130 (42.4 %) had neuropathy, and 110 (38.6 %) had nephropathy. The secondary prevention group had a significantly higher proportion of each of these three complications. Medications are presented in the final section of Table 1: a total of 177 (58. %) reported using acetylsalicylic acid, 220 (72.1 %) statin, 23 (7.5 %) ezetimibe, 1 (0.3 %) fibrate, and 218 (71.5 %) RAS inhibitor. Of these, the primary prevention group had markedly lower acetylsalicylic acid, statin, and ezetimibe use than the secondary prevention subgroup.

**Table 1:** Descriptive characteristics of 309 participants with longstanding type 1 diabetes

Characteristic	Total (n = 309)	Primary prevention (n = 204)	Secondary prevention (n = 105)	P value
Demographic				
Age (year)	65.7 ± 8.5	64.3 ± 8.3	68.5 ± 8.3	<0.001*
Duration of diabetes (year)	54.0 [51.0, 59.0]	53.0 [50.0, 57.0]	57.0 [52.5, 60.5]	<0.001*
Male [n (%)]	137 (44.5 %)	82 (40.4 %)	55 (52.4 %)	0.045*
Non-caucasian [n (%)]	13 (4.2 %)	10 (5 %)	3 (2.9 %)	0.40
Father’s age at death (year)	76.0 [66.0, 85.0]	76.0 [66.0,85.0]	76.5 [63.5, 84.3]	0.66
Mother’s age at death (year)	85.0 [77.0, 90.0]	85.0 [76.0,90.0]	84.5 [78.3, 90.0]	0.91
Parent with CVD [n (%)]	171 (56.4 %)	106 (53.3 %)	65 (62.5 %)	0.12
Sibling with CVD [n (%)]	68 (23.7 %)	38 (20.4 %)	30 (29.7 %)	0.08
Clinical				
BMI (kg/m^2^)	25.0 [23.0, 28.2]	24.9 [23.0,27.5]	25.7 [22.8, 29.1]	0.33
Systolic BP (mmHg)	128.8 ± 14.9	128.7 ± 14.6	129.0 ± 15.7	0.91
Diastolic BP (mmHg)	67.4 ± 8.9	67.8 ± 8.8	66.5 ± 9.1	0.37
History of hypertension [n (%)]	182 (60.3 %)	111 (56.1 %)	71 (68.3 %)	0.04*
Laboratory				
HbA1c (%)	7.5 ± 1.1 (58 mmol/mol)	7.4 ± 1.1 (57 mmol/mol)	7.7 ± 1.1 (61 mmol/mol)	0.04*
Total cholesterol (mmol/L)	4.1 ± 0.9	4.24 ± 0.92	3.95 ± 0.86	0.02*
Triglycerides (mmol/L)	0.80 [0.60, 1.05]	0.77 [0.60, 1.00]	0.83 [0.66, 1.23]	0.04*
HDL-C (mmol/L)	1.7 ± 0.5	1.79 ± 0.52	1.58 ± 0.49	0.002*
LDL-C (mmol/L)	2.0 ± 0.7	2.06 ± 0.70	1.92 ± 0.65	0.10
Complications				
Diabetic retinopathy [n (%)]^a^	193 (71.5 %)	116 (64.4 %)	77 (85.6 %)	<0.001*
Neuropathy (MNSI ≥3) [n(%)]	130 (42.3 %)	71 (34.8 %)	59 (57.3 %)	<0.001*
Nephropathy [n (%)]^b^	110 (38.6 %)	59 (31.7 %)	51 (51.5 %)	0.001*
Medications				
ASA [n (%)]	177 (58.0 %)	95 (47.5 %)	82 (78.1 %)	<0.001*
Statin [n (%)]	220 (72.1 %)	131 (65.5 %)	89 (84.8 %)	<0.001*
Ezetimibe [n (%)]	23 (7.5 %)	10 (5 %)	13 (12.4 %)	0.02*
Fibrate [n (%)]	1 (0.3 %)	1 (0.5 %)	0 (0.0 %)	0.47
RAS inhibitor [n (%)]^c^	218 (71.5 %)	141 (70.5 %)	77 (73.3 %)	0.60
ARB [n (%)]	92 (30.2 %)	55 (27.5 %)	37 (35.2 %)	0.16
ACEi [n (%)]	132 (43.3 %)	90 (45.0 %)	42 (40.0 %)	0.40

Data presented as mean ± SD, median [IQR], or n (%) unless otherwise indicated. Percentages were calculated from available data. P values for comparison were calculated using the student’s t-test, Mann–Whitney U test, or the χ^2^-test depending on variable distribution

*MNSI* Michigan neuropathy screening instrument, *BP* blood pressure; *HbA1C* glycated hemoglobin A1C, *ASA* acetylsalicylic acid, *RAS* renin-angiotensin system, *ARB* angiotensin II receptor blocker, *ACEi* angiotensin-converting-enzyme inhibitor

^a^Retinopathy as determined by most recent fundoscopy examination results

^b^Nephropathy defined by age-adjusted GFR <60 ml/min and/or ACR ≥2 mg/mmol

^c^RASi includes usage of at least one of ARB and ACEi

* Statistically significant p < 0.05

**Fig. 2:**
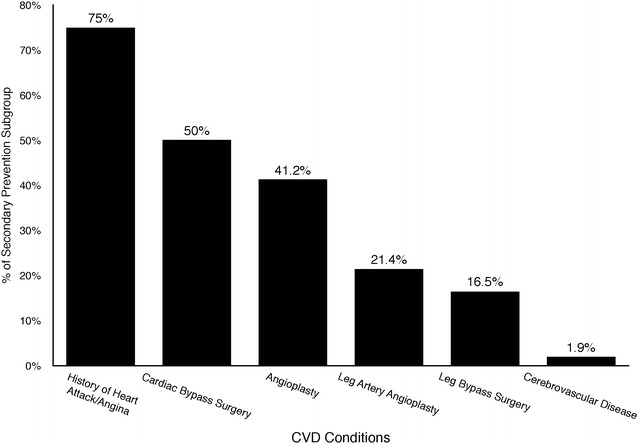
Prevalence of cardiovascular disease (CVD) conditions among the 105 participants with CVD

The distribution of cardiovascular conditions is presented in Fig. 2. Of the entire cohort, 105 (34 %) had cardiovascular conditions (and were included in the secondary prevention subgroup). Within this subgroup, 78 (75.0 %) reported history of heart attack or angina, 52 (50.0 %) had cardiac bypass surgery, 40 (41.2 %) had cardiac angioplasty, 16 (16.5 %) had leg bypass surgery, and 21 (21.4 %) had leg artery angioplasty, and 2 (1.9 %) had cerebrovascular disease. These two individuals also reported history of heart attacks, and one of them had a cardiac bypass surgery.

### Guideline adherence

Adherence to guideline recommendations is summarized in Table 2. The following results are reported for the cohort as a whole: Under the domain of lifestyle recommendations, 52.7 % of participants reported following a recommended diet, 96.8 % did not currently smoke, and 70.5 % reported current physical activity. Based on clinical exam and laboratory reports, 35.0 % had optimal HbA1C ≤7 % (53 mmol/mol), 47.8 % had blood pressure ≤130/80 mmHg, 57.7 % had LDL-C ≤2.0 mmol/L, and 50 % had optimal BMI <25 kg/m^2^. In terms of pharmacotherapy, 98.1 % of participants were eligible for RAS inhibitors and 72.5 % of these participants were using an ACEi or ARB. Finally, 72.1 % participants reported statin use. Among all participants, 62.5 % of guideline recommendations were met, which was the same between primary and secondary prevention subgroups. Agreement among each component of the adherence index was low (κ < 0.20), except for between statin use and LDL-C (κ = 0.29). The primary and secondary prevention subgroups had similar self-reported adherence to all the above recommendations except for physical activity and statin use. Specifically, compared to the secondary prevention subgroup the primary prevention subgroup had significantly higher proportion of participants who were physically active and lower prevalence of statin use.

**Table 2:** Measures of adherence to CVD prevention guidelines

Adherence category	Total (n = 309)	Primary prevention (n = 204)	Secondary prevention (n = 105)	P value
Lifestyle adherence				
Recommended diet^a^	159 (52.7 %)	105 (52.2 %)	54 (53.5 %)	0.84
Non-smoking	296 (95.8 %)	195 (95.6 %)	101 (96.2 %)	0.80
Physically active	217 (70.5 %)	154 (75.5 %)	63 (60.6 %)	0.007*
Clinical target attainment				
HbA1c ≤7.0 %	103 (35.0 %)	74 (38.1 %)	29 (29.0 %)	0.12
Blood pressure ≤130/80 mmHg^b^	76 (47.8 %)	53 (50.5 %)	23 (42.6 %)	0.35
LDL-C ≤ 2.0 mmol/L	150 (57.7 %)	95 (54.6 %)	55 (64.0 %)	0.15
Optimal BMI <25.0 kg/m^2^	149 (50 %)	101 (51.0 %)	48 (48.0 %)	0.62
Pharmacotherapy adherence				
RAS inhibitor^c^	221 (72.5 %)	144 (72.0 %)	77 (73.3 %)	0.80
Statin	220 (72.1 %)	131 (65.5 %)	89 (84.8 %)	<0.001*
Among participants Age <75^d^	190 (72.0 %)	117 (65.0 %)	73 (86.9 %)	<0.001*
Total adherence				
Median percentage of targets met	62.5 (50.0, 75.0)	62.5 (50.0, 75.0)	62.5 (50.0, 77.8)	0.93

Data presented as proportion n (%) achieving adherence, calculated as percentage of available data. P values for comparison are calculated using the Mann–Whitney U or χ^2^-test, depending on variable distribution

^a^Recommended diet measured by self-reported consumption of fruits and vegetables in addition to moderate consumption of dietary carbohydrates and fats

^b^Missing blood pressure: 150 (49 %) missing

^c^RASi includes usage of at least one of ARB and ACEi, percentages calculated out of only for participants who are eligible for RASi (see Methods)

^d^Statin use percentages calculated out of only participants under age 75 (n = 264), instead of whole cohort (n = 309)

* Statistically significant p < 0.05

In sensitivity analysis, results did not differ when alternate target thresholds were used for HbA1c, blood pressure, and BMI. Furthermore, when statin use was compared only in participants 75 years or younger, the primary prevention subgroup still had significantly lower statin prevalence than the secondary prevention subgroup (117 (65 %) vs 73 (86.9 %), p < 0.001).

### Factors associated with statin use

Univariable analyses demonstrated that female sex, hypertension history, higher BMI, lower LDL-C, lower HDL-C, lower total cholesterol, presence of at least one microvascular complication, nephropathy, retinopathy, absence of CVD history, acetylsalicylic acid use, and RAS inhibitor use were associated with the presence of statin use (Table 3). These variables, in addition to age and HbA1c, were used to create a multivariable model as shown in Table 4, which showed that only a higher cholesterol level (adjusted OR = 0.39 [0.25, 0.59] per unit increase in mmol/L, p < 0.001) and absence of CVD history (adjusted OR = 0.38 [0.15, 0.95] for membership in primary prevention subgroup, p = 0.04) were independently associated with lower statin use. Both factors remained significantly associated with statin after a stepwise selection process.

**Table 3:** Results of univariable logistic regression of variables associated with statin use in 309 participants

Variable	Odds ratio	95 % CI	P-value
Age (per year)	1.01	0.98, 1.04	0.58
Duration of diabetes (per year)	1.02	0.98, 1.06	0.38
Female	0.42	0.24, 0.71	0.001*
Parent with CVD	0.96	0.58, 1.59	0.86
Sibling with CVD	0.63	0.35, 1.13	0.12
History of smoking	1.28	0.77, 2.11	0.34
Physically active	0.86	0.49, 1.5	0.60
History of hypertension	1.69	1.01, 2.82	0.047*
BMI (per kg/m^2^)	1.1	1.03, 1.18	0.006*
HbA1c (per %)	1.09	0.86, 1.4	0.47
LDL-C (per mmol/L)	0.25	0.15, 0.41	<0.001*
HDL-C (per mmol/L)	0.44	0.26, 0.77	0.004*
Triglycerides (per mmol/L)	1.2	0.68, 2.15	0.53
Total cholesterol (per mmol/L)	0.35	0.24, 0.5	<0.001*
Microvascular complication^a^	2.21	1.19, 4.11	0.01*
Neuropathy	1.14	0.68, 1.89	0.62
Nephropathy	1.89	1.05, 3.39	0.03*
Retinopathy	2.04	1.16, 3.6	0.01*
Absence of CVD history	0.34	0.19, 0.63	<0.001*
ASA	3.44	2.04, 5.8	<0.001*
RASi	2.82	1.66, 4.81	<0.001*
ARB	1.89	1.05, 3.41	0.04*
ACEi	1.7	1.01, 2.86	0.046*

^a^Presence of at least one of neuropathy, nephropathy, or retinopathy, based on objective evidence

* Statistically significant p < 0.05

**Table 4:** Results of multivariable logistic regression, with statin use as the dependent variable

Variable	Odds ratio	95 % CI	P-value
Model 1			
Age (per year)	1.00	0.96, 1.05	0.86
Female	1.09	0.52, 2.32	0.81
History of hypertension^a^	0.81	0.36, 1.80	0.60
BMI (per kg/m^2^)	1.03	0.94, 1.13	0.56
HbA1c (per %)	1.19	0.85, 1.66	0.31
Total cholesterol (per mmol/L)	0.39	0.25, 0.59	<0.001*
Microvascular complication	1.55	9.64, 3.73	0.33
Absence of CVD history	0.38	0.15, 0.95	0.04*
ASA	1.80	0.89, 3.63	0.10
RASi	1.75	0.78, 3.93	0.17
Model 2			
Absence of CVD history	0.28	0.12, 0.63	0.002*
Total cholesterol (per mmol/L)	0.39	0.26, 0.56	<0.001*

237 (77 %) out of 309 possible observations had sufficient data to be included in this analysis

Model 1: All significant variables from univariable analysis included, with the exception of LDL and HDL, due to multicollinearity with total cholesterol. All microvascular complications were represented by presence of microvascular complication and ARB/ACEi were represented by RASi in the multivariable model. Age and HbA1c were forced into the model

Model 2: As a sensitivity analysis, stepwise selection process was used to decide on final model, with choice of variables the same as Model 1

^a^Sensitivity analysis using SBP instead of history of hypertension resulted in an adjusted odds ratio for absence of CVD history of 0.36 (95 % CI 0.11, 1.13, p = 0.07)

* Statistically significant p < 0.05

When the logistic regression was restricted to participants in the primary prevention subgroup, sex, age, total cholesterol, BMI, RAAS blockade, and aspirin use were associated with statin use in univariable analysis. When these were included in the multivariable model, only lower total cholesterol was significantly associated statin use.

## Discussion

In the cross-sectional analysis of 309 Canadians with longstanding type 1 diabetes uniformly considered to be at high CVD risk, we observed similar adherence to most general guideline recommendations between the primary and secondary prevention subgroups. However, against prevailing recommendations, the primary prevention subgroup had markedly insufficient statin use—approximately one-third odds relative to the secondary prevention subgroup. Such odds persisted in adjusted analysis to account for potential confounding variables: age, sex, hypertension history, greater BMI, higher HbA1c and total cholesterol, presence of microvascular complications, and acetylsalicylic acid and RAS inhibitor use.

### Suboptimal self-reported statin use in the context of the literature

Suboptimal statin use has been commonly reported in a variety of study populations, and is associated with financial, drug-related, health system-related, condition-related, and patient and physician-related factors [17]. Statin use in large clinical trials typically ranges from continuation rates of 70–90 % of trial participants [32–34]. Cross-sectional studies in patients with diabetes in real-world clinical settings have reported much lower rates, generally approximating 50 % prevalence of statin use [35–37]. Most of these studies have been conducted in the context of type 2 diabetes, though some limited data suggests even lower statin use in type 1 diabetes with estimated prevalence below 50 % [38]. Our study addresses this paucity of evidence on statin use in type 1 diabetes by studying individuals with longstanding diabetes who are at high CVD risk and uniformly require statin use for CVD prevention. It is encouraging to note that statin prevalence in our participants (72.1 %) exceeds that in most observational studies, and even approaches the high rates observed in statin clinical trials. Nonetheless, the concern remains that there was a key disparity between primary and secondary prevention subgroups, with about 20 % lower statin use in those without a history of CVD. This implies a significant care gap in the primary prevention of CVD in patients with type 1 diabetes which puts these patients at high risk of a first CVD incident [19, 20]. To estimate the potential clinical implication of this care gap, a simulation study demonstrated that a 25 % increase in statin prevalence in a primary prevention cohort is predicted to avert up to 53 % more CVD-related deaths over 10 years [39]. We therefore hypothesize that increasing statin use among type 1 diabetes patients without a history of CVD to approximate that observed in patients with CVD could represent a substantial strategy to reduce CVD mortality. From a public health perspective, our results suggest that targeting improved statin use in longstanding type 1 diabetes presents an opportunity to decrease the CVD incidence and mortality.

### Comparison to literature for other general guideline recommendations

Regarding other CVD prevention recommendations, adherence in our participants was similar to that in other cross-sectional studies in outpatient diabetes settings [35–37]. In fact, it is reassuring that as a whole, our participants had HbA1c, BMI, blood pressure, and lipid measures which were close to or better than guideline recommendations. Remarkably, our cohort had a RAS inhibitor prevalence of 72.5 % amongst eligible participants–similar between primary and secondary prevention subgroups–which approximates that previously found in type 1 diabetes populations [40]. Interestingly, high ACEi and ARB use was uniform between the two subgroups despite greater prevalence of hypertension and nephropathy in the secondary prevention subgroup. This is in contrast to lower statin use in the primary compared to secondary prevention subgroup, even though the two subgroups had similar levels of LDL-C. Perhaps this phenomenon suggests a strong recognition by clinicians and patients of the protective benefits of RAS inhibition, and in contrast, an incorrect but prevailing clinical view that statin use should be limited to those with CVD or dyslipidemia. This data strongly supports the notion that clinicians and patients may not appreciate long diabetes duration as a significant CVD risk factor and are thus reluctant to use statin for primary prevention—a view which has been disproven by the results of many large studies [7–9].

### Study limitations

While this study is the first to investigate attention to clinical practice guidelines and self-reported statin use in the context of longstanding type 1 diabetes, and it used mixed methods of data acquisition including self-report, validated questionnaires, and laboratory measures, we recognize some limitations and sources of potential bias. First, our investigation of extreme diabetes duration carries a risk of selection bias—specifically, incidence-prevalence (survival) bias—whereby participants may have had better life-long management of CVD risk compared to those who did not survive to 50 years diabetes duration. However, though the magnitude of adherence and CVD prevalence may be affected by such incidence-prevalence bias, it is unlikely that it would affect the observed association of statin use and the primary and secondary prevention subgroups. Secondly, we acknowledge the risk of recall bias and consequent misclassification error, though we expect these to be small in magnitude given the discernible nature of CVD and current medication use, and we emphasize that such recall bias is non-differential between our analytical subgroups and that the odds ratios presented are unlikely to be influenced by this bias. Third, ascertainment of cerebrovascular disease events may have been incomplete, but the low prevalence of these events is in keeping with known rates from epidemiological study of type 1 diabetes [41]. Fourth, while we believe that our analysis has considered the most fundamental confounders through adjustment, there remains the possibility of unmeasured and residual confounding. For instance, other studies have found retirement and female sex to be associated with lower adherence to statin use and prescription, respectively, [42, 43]; these variables may require further study in our cohort. Fifth, as this study was a secondary analysis, we only determined prevalence of statin use rather than a direct measure of medication adherence such as proportion of days covered by statin prescriptions or reasons—such as medication side-effects—for statin non-use. Finally, our results are specific to those with longstanding type 1 diabetes and may not extend to T2DM or older adult populations without diabetes.

## Conclusions

Adherence to cardiovascular protection guidelines—especially statin use—is of fundamental clinical importance because under-treatment in high-risk individuals can worsen cardiovascular disease risk and increase the burden to the healthcare system [17, 23]. This study supports that Canadians with longstanding type 1 diabetes have relatively high self-reported adherence to guidelines and statin prevalence as a whole, but there is inappropriately lower statin use for CVD primary prevention than secondary prevention. In view of this apparent clinical disregard for longstanding diabetes as a fundamental CVD risk factor, our results may serve to encourage improved adherence to evidence-based recommendations for primary prevention of CVD with statins in this population. Future research with this unique cohort should focus on elucidating the causes of suboptimal statin use, and interventions should address statin disparity between primary and secondary CVD prevention in patients with longstanding type 1 diabetes.
